# Development and validation of the activities and participation children and adolescents –neck (APCAN) measure

**DOI:** 10.1186/s41687-023-00648-x

**Published:** 2023-10-30

**Authors:** Devashish Tiwari, Keshrie Naidoo, Emily Z. Eddy, Naseem Chatiwala, Maninderjit Kaur

**Affiliations:** Department of Physical Therapy, MGH Institute of Health Professions, 36, 1st Ave, 02129 Boston, MA USA

## Abstract

**Background:**

Neck pain is the fourth leading cause of years lost to disability in children warranting a comprehensive assessment of neck pain and its impact on activities and participation. Hence, the purpose of this study was to develop a new measure (i.e., Activities and Participation Children and Adolescents -neck [APCAN]) specific to evaluating activity limitation and participation restrictions in children and adolescents and to establish its content validity.

**Methods:**

Development and content validation of the APCAN was completed in four steps: (1) item development, (2) item evaluation by content experts, (3) content validity calculation, and (4) cognitive testing via interviews to ensure readability and comprehension of the items on the APCAN.

**Results:**

An initial pool of 52 items was created that was revised to 20 items after modified Delphi process and cognitive interviews. Each item was rated on a 0–10 numeric rating scale (0 = not difficult at all, 10 = extremely difficult) with higher scores indicating higher perceived disability secondary to neck pain. All 20 items retained the content validity ratio critical value and the overall content validity index was 0.88 indicating excellent content validity.

**Conclusion:**

The APCAN provides an easy to use, comprehensive assessment of functional limitations associated with neck pain in children.

**Supplementary Information:**

The online version contains supplementary material available at 10.1186/s41687-023-00648-x.

## Background

Neck pain among children and adolescents is a rapidly growing health concern [1–3]. Annual prevalence of neck pain in children in the United States from all causes is as high as 71.5% [4, 5]. The World Health Organization (WHO) has identified neck pain as the fourth leading cause of years lost to disability among the 10–14 years-old population [6]. A recent increase in the use of smart devices such as phones, hand-held video game consoles, and tablets has resulted in an increase in the adoption of poor postures sustained over long periods. The resultant increased strain on the neck muscles (commonly known as text neck), has contributed to the increased overall incidence of neck pain in children [2, 7, 8].

In children and adolescents, neck pain demonstrates a strong association with increased perceived disability, [2, 9] thereby negatively affecting quality of life, environment exploration, and participation in the community. Additionally, neck pain results in increased missed school days, poor academic performance, and poor sleep, resulting in negative health behaviors, [10] such as being more irritable and feeling alienated [2]. Subsequently, children and adolescents with neck pain may miss crucial opportunities for skill development, social interaction, and meaningful activity participation [10, 11] Considering the substantial negative impact of neck pain on health behaviors in children, it is vital to screen for and comprehensively examine neck pain and its impact on body functions, activities, and participation in this population using valid Patient-reported outcome measures (PROM).

PROM capture the impact of a disease and/or intervention on the patient. They provide an excellent medium to steer healthcare toward a patient-centered care model through integrated care [12]. Specifically, in the pediatric population, children between 3 and 7 years can respond to simple PROM (for example, the Faces Pain Scale), [13] whereas 7–8 years old have sufficient cognitive skills to respond appropriately to systematic questioning [14, 15]. Allowing the child to self-report pain leads to better assessment and treatment design to support improved outcomes in medical or rehabilitative models of care [16]. Information obtained directly from the child is instrumental in designing focused rehabilitation interventions to address pain, and address the environmental and social contexts in which (1) the neck pain arises and/or (2) children and adolescents are avoiding, to ensure healthcare providers are considering all relevant factors to support function.

Previous studies have reported acceptable reliability of PROM for children 8 years and older [17]. Currently, there is a clear lack of PROM for neck pain assessment in the pediatric population as the commonly used neck pain PROM like the Neck Disability Index and Whiplash Disability Questionnaire were designed for adults and they do not cover age-appropriate activities, and demonstrate poor validity for children and adolescents [18, 19]. There are some pediatric measures such as the Adolescent Pediatric Pain Tool, [20] Pediatric Pain Questionnaire, [21] and The National Institutes of Health (NIH) Patient Reported Outcomes Measurement Information System (PROMIS) pediatric pain interference scale [22] that focus on pain assessment in children and youth. However, these measures are generic, not specific to neck pain, do not cover the interaction of how the pain restricts participation and limits activities across multiple environments, and have not been used to evaluate neck pain in this population [13, 23]. Additionally, existing measures contain items that involve constructs like walking, running and, stair climbing which may not be areas of significant functional limitations for children who have neck pain. Compared to generic measures, condition-specific measures may provide better relevance and responsiveness and demonstrate the potential to differentiate patient groups by clinically predominant symptoms or health concerns [24].

It is imperative that measures capture the perception of disability due to neck pain in children and adolescents to understand the specific role of neck pain in activity limitation and participation restriction. The International Classification of Functioning, Disability and Health- Children and Youth (ICF-CY) model provides a guiding framework and shared language for conceptualizing and identifying children and youth who experience any delay or impairment of body functions, structures, and skill acquisition [25]. The ICF-CY model has a significant impact on the healthcare and education services globally and is used by clinicians, educators, policymakers, and researchers to document characteristics of health and functioning in children and youth [26]. Designing a PROM that uses the ICF-CY constructs and terminology will help clinicians such as physical and occupational therapy practitioners to establish age-appropriate therapeutic goals for the child.

Lack of valid age-specific measures in school age children limits the ability of clinicians to make well-informed clinical decisions and set appropriate patient-centered goals [2]. Currently, no measure exists to specifically assess the influence of neck pain on body function, activities, and participation in children and adolescents. Hence, the purpose of this study was to develop a new measure (i.e., Activities and Participation Children and Adolescents -neck [APCAN]) specific to evaluating activity limitation and participation restrictions in children and adolescents and to establish its content validity.

## Methods

Content validation process followed the COnsensus based Standards for the selection of health status Measurement INstruments (COSMIN) guidelines [27] Development and content validation of the APCAN was completed in four steps: (1) item development, (2) item evaluation by content experts, (3) content validity calculation, and (4) cognitive testing via interviews to ensure readability and comprehension of the items on the APCAN. Information on existing neck pain measures and details on their content comparison was informed by our previously published work [28]. It was noteworthy that none of the measures were valid for children and adolescents.

### Item development

The first step in developing a patient-reported measure is to identify the constructs intended to be assessed by the measure [15]. Our previously published work to systematically compare content across existing measures of neck pain informed this step. Content from these existing measures was utilized to identify some potential items to be included on the APCAN [28]. Additionally, a comprehensive pool of items related to activities and participation was further developed by the team following an exhaustive review of the ICF-CY model [25]. The items were written in child-friendly language and kept at 3rd grade reading level [29]. As recommended by previous research, the items of the APCAN were positively phrased to make the measure appropriate for children [30].

### Linkage to ICF-CY

Two experts from the study team linked each item from the APCAN to the ICF-CY categories. ICF-CY follows a classification system that is organized based on hierarchy and inter-relatedness of levels. The ICF-CY consists of a section on functioning and disability that includes body functions and structures and activities and participation, and a section on contextual factors that includes environmental and personal factors.

### Expert consultation

A measure has content validity if “it covers all parts of the universe of content and reflects the importance of each part” [31]. A modified Delphi process was used for content validation. Modified Delphi process is a technique to obtain the most reliable consensus from a group of experts [32]. Ten clinical experts in different fields of practice (physical therapy, occupational therapy, and nursing) were recruited via email to participate in the modified Delphi process [31]. The experts were selected based on their content, clinical, or research expertise in pediatric neck pain. The experts participated in two rounds of modified Delphi process [33, 34]. A detailed explanation of the different items on the list and instructions on how to score the items was provided to assist the experts in their rating. Experts were asked to independently rate each item as “essential”, “useful but not essential” or “not necessary” as recommended previously by Lawshe [35]. The experts were requested to provide a rationale for their responses. The experts had three weeks to complete the first round and a reminder email was sent after two weeks. All responses were documented for the next step of content validation. The same procedure was followed for the second round of review.

### Content validation

Two research team members completed all analysis. To determine content validity, the content validity ratio (CVR) was calculated using Lawshe’s formula CVR = (ne – N/2)/N/2 where “ne” is the number of experts identifying an item as “essential” whereas N is the total number of experts [34, 36]. CVR values range from − 1 to + 1. A CVR value above zero indicated that over half of the experts agreed the item was “essential”. Lawshe and Schipper’s table of critical values was used to determine the critical value of CVR (CVR_critical_) to eliminate chance agreement between experts. Items were retained in their original form if the CVR values were above the CVR_critical_ (Table 1) [35]. Once all rounds of review were complete, the content validity index (CVI) was calculated to obtain a numeric value of the content validity of the measure [34, 36]. The CVI was calculated as the mean of the overall CVRs for all the items included in the final measure. A CVI value of > 0.8 was considered an indicator of good content validity [34].

**Table 1:** Content validity ratio values for individual items after two rounds of modified Delphi process

Item number	ICF CY category	Item details	Content validity ratio
1	Body function	Within the last week, I can pay attention to what I am doing for more than 30 min. For example: Watching TV, leisure reading, homework/classwork	0.8
2	Body function	I sleep well at night, and I feel well rested even when my neck hurts.	1
3	Body structure, activity	Right now, I can move my neck (looking behind me, looking up/down or over my shoulder).	1
4	Body function	I can lift my arms over my head without neck pain.	0.8
5	Activity	I can brush my teeth, take a shower, and wash my hair without neck pain.	0.8
6	Activity	I can put on a shirt/sweatshirt easily and without neck pain.	0.8
7	Activity	I can sit on a chair (at home, in the classroom etc.) comfortably for more than 30 min.	1
8	Activity	I can bend down to pick up toys/things from the floor.	0.8
9	Activity, participation, environmental	I can do these outdoor things just the way I do them when I do not have pain. For example, ride my bike/scooter/skateboard, climb on a slide, play on a play structure, and jump on a trampoline. If your choice is not in the examples, you can write your own activity of choice below. Other preferred activity of choice: ______	0.8
10	Activity	I can play with toys/board games/puzzles/do arts and crafts while sitting on a chair/floor like I usually do.	1
11	Activity	I can do these technology related things easily (For example Talk and/or text on the phone while holding the phone in my hands, play video games, use my tablet/I-pad). If your choice is not in the examples, you can write your own activity of choice below. I use technology most often for:	1
12	Activity	I can run/hop/skip/jump.	1
13	Activity	I can throw a ball (For example, Football, baseball, basketball) as far as I want.	1
14	Activity, participation	I can do these things as I usually do. Select all that apply: ● Write, type in class/home. ● Classroom or leisure reading Others:	0.8
15	Activity	I can ride in a car/school bus.	0.8
16	Activity	I can stand/walk for up to 10 min with my backpack on my shoulders.	0.8
17	Participation	I can help with household chores (for example, wash dishes, use a broom, vacuum, make my bed, and clean my room). If your choice is not in the examples, you can write it below. The chores I do most often:	1
18	Participation	I can participate in recreational sports like swimming, gymnastics or other sporting activity of my choice for as long as I want. Specify (other sport of choice):	0.8
19	Participation	I can attend school regularly.	1
20	Participation	I can keep up with friends in gym class/siblings at home.	0.8

### Cognitive interviewing

Approval from the Institutional Review Board of the MGH Institute of Health Professions was obtained (approval number #2022P001971). Children and adolescents aged 8–18 years old who speak English as a primary language and currently experiencing neck pain or with a history of neck pain were recruited via a flyer posted to the researchers’ social media accounts. After obtaining written consent from the parents and assent from the children, cognitive testing of the APCAN was performed. Cognitive interviewing is a form of qualitative interviewing used to obtain insights about a respondent’s thought process as they read or hear an item, and as they respond to a question. The purpose of cognitive interviewing and testing is to explore whether children understand the questions consistently in the way intended by the researchers [15]. Cognitive interviewing was completed by two members of the research team using 1:1 interviews on Zoom with children currently experiencing or with a history of neck pain. One-on-one interviews, as opposed to focus group interviews were used to control for bias (such as peer pressure) which can occur in a group setting with children [15]. Additionally, it is difficult for the younger children to stay attentive to the questions in a focus group [15]. Participants were provided with a $15 gift card for participating.

Characteristics of children who participated in the cognitive interview are reported in Table 2. During round one of cognitive interviewing, researchers conducted 1:1 interview with three children ranging in age from 14 to 17 years. Two participants had experienced neck pain within the last 18 months, and one participant was currently experiencing neck pain. A copy of the measure was provided to the participant for review prior to the interview. During the interview, a research team member read the items from the measure to the participant and the item was pasted in the Zoom chat. The participant was then asked to think out loud and describe their thought process and how they interpreted the survey response and formulated a response [37] In addition, each participant was asked to suggest any modifications/additions to the items. Responses were audio recorded using a digital audio recorder, transcribed, and deidentified.

**Table 2:** Characteristics of children who participated in cognitive interviews

Participant number	Interview date	Age	Gender	History of neck pain
1	1.9.23	17	F	Currently experiencing neck pain
2	1.13.23	14	M	9–12 months prior to interview
3	1.13.23	16	M	12–18 months prior to interview
4	3.7.23	10	M	6–7 months prior to interview
5	3.9.23	9	F	10–12 months prior to interview
6	3.9.23	10	M	Currently experiencing neck pain

After three interviews, researchers reviewed the transcripts and agreed on the items that required modification. Once the changes were finalized, the revised measure was subjected to a second round of cognitive interviews. Researchers again conducted a total of three 1:1 interview with children with a history of neck pain ranging in age from 10 to 14. After interviews four and five, minor modifications were made to three items (Table 3). After interview six, no further changes were warranted, and the interviews were stopped [38]. This process allowed researchers to ensure that survey items met the requirements for face validity that the questions were unambiguous and clear to a child.

**Table 3:** Revision of Activities and Participation Children and Adolescents – Neck (APCAN) after two rounds of cognitive interviews

Item number	ICF CY code	Item details	Revised item after first round of cognitive interviews	Revised item after second round of cognitive interviews
1	b140	Concentrating/focusing on an activity of choice for more than 30 min *Preferred*: Watching TV, leisure reading *Non-preferred*: Doing homework/classwork	Within the last week, I can pay attention to what I am doing for more than 30 min. For example: Watching TV, leisure reading, homework/classwork	
2	b134	Falling asleep/staying asleep	I sleep well at night and I feel well rested even when my neck hurts.	
3	b780, s710	Moving your neck (for example, looking behind you, looking up/down or over your shoulder).	Right now, I can move my neck (looking behind me, looking up/down or over my shoulder).	
4	b730	Lifting the arms over your head	I can lift my arms over my head without neck pain.	
5	d445, d520, d 150	Brushing teeth, grooming, brushing or washing hair, taking a bath/shower	I can brush my teeth, take a shower, and wash my hair without discomfort.	I can brush my teeth, take a shower, and wash my hair without neck pain
6	d540	Dressing your upper body (for example: putting on a sweatshirt)	I can put on a shirt/top/sweatshirt easily and without neck pain.	I can put on a shirt/sweatshirt easily and without neck pain.
7	d140	Sitting in a chair (home, classroom etc.) for more than 30 min	I can sit on a chair (at home, in the classroom etc.) comfortably for more than 30 min.	
8	d455, d410	Bending down to pick up toys/objects from the floor	I can bend down to pick up toys/things from the floor.	
9	d475, e115, d455	Participating in an age-appropriate activity. Select all that apply: • Riding my bike/scooter/skateboard • Climbing on a slide • Playing on a play structure Jumping on a Trampoline	I can do these outdoor things just the way I do them when I do not have pain. For example, ride my bike/scooter/skateboard, climb on a slide, play on a play structure, and jump on a Trampoline. If your choice is not in the examples, you can write your own activity of choice below. Other preferred activity of choice: ______	
10	d920	Playing with toys/board games/puzzles/doing arts, crafts	I can play with toys/board games/puzzles/do arts, crafts while sitting on a chair/floor like I usually do.	I can play with toys/board games/puzzles/do arts and crafts while sitting on a chair/floor like I usually do.
11	e125, d110, d360	Using a hand held device. Select all that apply: • Talking and/or texting on the phone • Playing video games Operating tablet/I-pad	I can do these technology related things easily (For example Talk and/or text on the phone while holding the phone in my hands, play video games, use my tablet/i-pad). If your choice is not in the examples, you can write your own activity of choice below. I use technology most often for:	
12	d455	Running/hopping/skipping/jumping	I can run/hop/skip/jump.	
13	d445	Throwing/catching/bouncing a ball	I can throw a ball (For example, Football, baseball, basketball) as far as I want.	
14	d170, d345, d155 d166	Performing the following activities. Select all that apply: ● Writing ● Typing in class/home ● Classroom or leisure reading ● Using fork and knife Others: please specify	I can do these things as I usually do. Select all that apply: ● Write, type in class/home ● Classroom or leisure reading Others:	
15	d470	Riding in the school bus/car and/or driving a car	I can ride in a car/school bus.	
16	d430	Carrying books/ school bag/backpacks/toys	I can stand/walk for up to 10 min with my backpack on my shoulders.	
17	d640	Helping with household chores. Select all that apply: • Washing dishes • Using a broom • Vacuuming • Making bed • Cleaning room Others (please specify):	I can help with household chores (for example, wash dishes, use a broom, vacuum, make my bed, and clean my room). If your choice is not in the examples, you can write it below. The chores I do most often:	
18	d220, d920	Participating in recreational sports like swimming, gymnastics or other sporting activity of choice **Specify**:	I can participate in recreational sports like swimming, gymnastics or other sporting activity of my choice for as long as I want **Specify (other sport of choice)**:	
19	d830, d820, d815	Attending school/preschool regularly	I can attend school regularly.	
20	d920	Keeping up with friends in the gym class/siblings at home	I can keep up with friends in the gym class/siblings at home.	I can keep up with friends in the gym class/siblings at home.

## Results

The first draft of APCAN had 52 potential items (Appendix 1). This draft was used for the modified Delphi process. All experts completed both rounds of modified Delphi process. Based on the number of experts (n = 10), the CVR_critical_ was set at 0.62 to retain items [35]. Items with the CVR less than 0.62 but more than 0.5 were modified and the items with CVR less than 0.5 were eliminated. After the first review, 14 items were eliminated, and 11 items were merged/modified resulting in 29 items that were sent for a second round of review. Following the second round, based on the expert feedback and the CVR_critical_ values, one item was eliminated, and eight items were merged with the other items belonging to the same construct and the measure was revised to 20 items (Table 4). All 20 items retained the CVR_critical_ values. The details of the CVR ratings can be found in Table 1. The CVI of the items retained in the measure was calculated and was found to be 0.88 that indicated good content validity [34].

**Table 4:** Activities and Participation Children and Adolescents – Neck (APCAN)

Item number	ICF CY category		Item details		
1	Body function		Within the last week, I can pay attention to what I am doing for more than 30 min. For example: Watching TV, leisure reading, homework/classwork		
2	Body function		I sleep well at night, and I feel well rested even when my neck hurts.		
3	Body structure, activity		Right now, I can move my neck (looking behind me, looking up/down or over my shoulder).		
4	Body function		I can lift my arms over my head without neck pain.		
5	Activity		I can brush my teeth, take a shower, and wash my hair without neck pain.		
6	Activity		I can put on a shirt/sweatshirt easily and without neck pain.		
7	Activity		I can sit on a chair (at home, in the classroom etc.) comfortably for more than 30 min.		
8	Activity		I can bend down to pick up toys/things from the floor.		
9	Activity, participation, environmental		I can do these outdoor things just the way I do them when I do not have pain. For example, ride my bike/scooter/skateboard, climb on a slide, play on a play structure, and jump on a trampoline. If your choice is not in the examples, you can write your own activity of choice below. Other preferred activity of choice: ______		
10	Activity		I can play with toys/board games/puzzles/do arts and crafts while sitting on a chair/floor like I usually do.		
11	Activity		I can do these technology related things easily (For example Talk and/or text on the phone while holding the phone in my hands, play video games, use my tablet/i-pad). If your choice is not in the examples, you can write your own activity of choice below. I use technology most often for:		
12	Activity		I can run/hop/skip/jump.		
13	Activity		I can throw a ball (For example, Football, baseball, basketball) as far as I want.		
14	Activity, participation		I can do these things as I usually do. Select all that apply: ● Write, type in class/home. ● Classroom or leisure reading Others:		
15	Activity		I can ride in a car/school bus.		
16	Activity		I can stand/walk for up to 10 min with my backpack on my shoulders.		
17	Participation		I can help with household chores (for example, wash dishes, use a broom, vacuum, make my bed, and clean my room). If your choice is not in the examples, you can write it below. The chores I do most often:		
18	Participation		I can participate in recreational sports like swimming, gymnastics or other sporting activity of my choice for as long as I want. Specify (other sport of choice):		
19	Participation		I can attend school regularly.		
20	Participation		I can keep up with friends in gym class/siblings at home.		

Following the modified Delphi process, the APCAN comprised 20 items that target various aspects of activity limitation and participation. Each item was rated on a 0–10 numeric rating scale (0 = not difficult at all, 10 = extremely difficult) with higher scores indicating higher perceived disability secondary to neck pain. Content mapping was performed for each item using the ICF-CY model (Table 4).

After three cognitive interviews, 20 items were revised due to a lack of clarity. For example, item 1 “Concentrating/focusing on an activity of choice for more than 30 minutes. Preferred: Watching TV, leisure reading. Non-preferred: Doing homework/classwork” was reworded to “Within the last week, I can pay attention to what I am doing for more than 30 minutes. For example: Watching TV, leisure reading, homework/classwork” (Table 3).

After the second round of cognitive interviews with three more children, three items were reworded to improve clarity. For example, item 5 “I can brush my teeth, take a shower, and wash my hair without discomfort” was reworded to “I can brush my teeth, take a shower, and wash my hair without neck pain” to maintain consistent language. After the third interview in the second round of cognitive interviews, item revision was no longer required. The last participant demonstrated an understanding of all items. No further interviews were conducted.

## Discussion

The current study aimed to develop and establish the content validity of a new PROM for assessing activity and participation restriction in children and adolescents. APCAN was developed using an evidence-based iterative and systematic process. The final measure has 20 items where each item is rated on a numeric rating scale ranging from 0 to 10. It was intentionally decided to not utilize a Likert scale rating for this measure. Previous studies have highlighted that children often experience difficulty in interpreting the middle point (e.g. moderate) on Likert ratings [39]. Additionally, children have shown to demonstrate difficulty in quantifying the differences between response options (e.g. occasionally, almost never) [40]. On the other hand, Numeric rating scale (ranging from 0 to 10) has been well-established as a reliable and valid measure of pain intensity in children and adolescents [41].

While the numeric rating scale might not be useful for younger children, evidence suggests that children over 8 years of age can understand and reliably use the numeric rating scale. This was also confirmed during cognitive interviews with children [42].

To make clinical decision making around degree of activity limitation easier, this measure allows for a summary score that can be calculated by adding the individual scores on each item. Scores could range from 0 (no disability) to 200 (maximum disability). This method allows for an initial approximation of the severity of limitation that could be used by clinicians on initial evaluation and reassessments to gain a broader perspective on the child’s problem [43]. However, items on this measure can also be examined individually to get a more in-depth information on the child’s limitation, since each item on the measure has a unique contribution towards the overall activity and participation limitation. For e.g., a child’s overall score on the APCAN of 10 out of 200, provides an initial indication that the overall severity of the impact of neck pain on activity and participation is potentially low. However, the overall score does not provide information on where this limitation stemmed from and would benefit by further exploration of individual items. It is likely that the child had maximum difficulty (10/10) in performing one specific activity, or moderate difficulty across multiple activities. This individual item exploration will provide clinicians a sound framework to design goals for management.

### Face and content validity of APCAN

Content of a PROM is the most critical element to ensure the quality of a measure. Since a measure with poor content validity cannot be improved with any statistical manipulation, content validation must be performed with sufficient scientific rigor prior to any other psychometric evaluation [44]. Several steps were taken to ensure a systematic and scientifically rigorous approach towards development of this measure. The ICF framework is universally recognized and accepted as a theoretical framework for health and provides a standardized coding system [45]. The ICF-CY has been used extensively for analysis of content across PROM [28, 46]. Additionally, COSMIN guidelines were followed while developing and establishing the content validity (Appendix 2) [27]. Based on COSMIN recommendations, good face and content validity were attained through a thorough review of the ICF-CY model, a review of existing measures specific to neck pain, inclusion of experts from multiple disciplines that closely work with patients with pediatric neck pain, and the use of a systematic process of obtaining stakeholder input to revise and refine the measure (Appendix 2). While expert input is critical to develop any measure, the type of expertise is crucial. Every item on the draft measure was further improved in language and comprehensibility after interviews with children currently experiencing neck pain or with a history of neck pain.

CVI values above 0.70 have been recommended in literature as adequate evidence of content validity and CVI values above 0.80 are recommended to establish good content validity (Appendix 2) [47, 48]. The APCAN demonstrates strong content validity with a CVI of 0.88.

### Comprehensiveness

The APCAN was formed to address all components of COSMIN recommendations to ensure comprehensiveness, including use of appropriate qualitative data, skilled interviewers, use of interview guides, and interviewing till attainment of data saturation. Appendix 2 highlights specific details on the use of COSMIN to ensure comprehensiveness. Comprehensiveness of APCAN as a functional scale for pediatric neck pain assessment can be attributed to several factors. First, APCAN assesses both neck pain and activity/participation limitations in children. Assessment of pain is a broad construct, and outcome measures that assess pain intensity alone are inadequate for assessing functional constructs of pain [49]. Since functional recovery is essential for both acute and chronic pain and the most important patient identified goal, [50] it is crucial to include the perceived impact of pain on activities of daily living separate from the focus on the pain intensity.

Second, APCAN provides a well-rounded representation from different domains of the ICF-CY allowing for a comprehensive assessment of activity limitation and participation restriction in children and adolescents. This measure was designed intentionally to focus on the impact of pain on activities and participation categories to help rehabilitation professionals identify impairments aligned to function and better address patient goals. All the items on the APCAN were successfully linked to the ICF-CY categories [25]. Additionally, the ICF-CY model focuses on the enablement perspective that further strengthens clinical reasoning [51]. Enablement models highlight the global view of a child’s performance in various contexts [51]. Additionally, the enablement model takes into account health rather than dysfunction and focuses on determining goals and intervention planning based on the child’s desire to participate in life roles [51] The APCAN uses the enablement model to provide context for children to select and add activities that are a priority to the child.

### Advantages of the measure

This measure offers several advantages over existing pain measures in the pediatric population. Currently, there is no available age-appropriate measure that specifically assesses the impact of neck pain on activities and participation. While PROMIS pain interference scale assesses generic aspects of pain, it does not specifically examine the impact of neck pain in children and its measurement properties are not established for children with neck pain. Particularly, some items in this measure such as “I needed help walking when I was in pain” and “I walked carefully when I was in pain” may not be applicable to children with neck pain. Additionally, questions in the PROMIS measure have a negative formulation. Evidence suggests that use of negatively framed questions are not preferred in children as these can force the respondent to make a negative statement to provide a positive answer [30, 52].

Existing outcomes measures that do assess neck pain were not designed specifically for the child and adolescent population and do not use the ICF-CY model [53]. Additionally, these measures show a misalignment between the symptoms expressed by the patients with neck pain and the content of the questionnaires [54].

Finally, while using parent reported measures, it is observed that parents may either overestimate or underestimate the impact of pain in children due to the parent’s personal characteristics and contextual factors [55]. This may lead to a mismatch between the clinician’s, child’s, and parent’s perspective on creating meaningful and appropriate goals [55, 56]. Given that children above the age of 8 can reliably use self-report measures, [17, 42]. APCAN, as a self-report measure, has the potential to accurately assess the pain and functional limitations of children.

Creating goals that are important and meaningful to the child is crucial to behavior change, vital to attaining improvements in goal performance [57] APCAN provides room for children to add specific activities of their choice in four questions (Q. 9, 11,14 and 17). The inclusion of activities specifically selected by children provides a strong framework for creating collaborative goal setting between the child, rehabilitation professional, family, and other professionals thereby facilitating family-centered care [56, 58].

### Limitations and future directions

Although this measure was developed after removing and collapsing several items from the original item pool, it still has 20 items, which could be considerably lengthy. Future studies to develop a computerized adaptive version that self-selects items based on responses could be helpful in reducing the administration of the measure. While the APCAN provides a good starting measure that is immediately available for clinicians to use in children 8–18 years of age with neck pain, we recommend testing of this measure in different population subsets before establishing generalizability. Cognitive interviewing was limited to children who speak English as a primary language. Future research should include translating and cross-culturally adapting the neck pain measure to other languages. Finally, further research on examining factor structure using exploratory factor analysis, establishing internal consistency and construct validity using Rasch analysis are needed to strengthen the APCAN.

## Conclusion

The APCAN fills the much-needed gap for a comprehensive assessment of children with neck pain. It provides an easy to use and comprehensive assessment of limitations in function associated with neck pain in children. Clinicians can gather meaningful information from APCAN to identify functional limitations, create therapeutic goals and monitor effectiveness of targeted rehabilitation interventions.

### Electronic supplementary material

Below is the link to the electronic supplementary material.

Supplementary Material 1

Supplementary Material 2

Supplementary Material 3

Supplementary Material 4

## Data Availability

Not Applicable.

## Data Availability

Data sharing is not applicable to this article as no datasets were generated or analysed during the current study.

## References

[1] Cox J, Davidian C, Mior S (2016). Neck pain in children: a retrospective case series. J Can Chiropr Association.

[2] Fares J, Fares M, Fares Y (2017). Musculoskeletal neck pain in children and adolescents: risk factors and Complications. Surg Neurol Int.

[3] Smith L, Louw Q, Crous L, Grimmer-Somers K (2009). Prevalence of neck pain and headaches: impact of computer use and other associative factors. Cephalalgia.

[4] King JA, McCrea MA, Nelson LD (2020). Frequency of primary Neck Pain in mild traumatic Brain Injury/Concussion patients. Arch Phys Med Rehabil.

[5] Manchikanti L, Abdi S, Atluri S (2012). American Society of Interventional Pain Physicians (ASIPP) guidelines for responsible opioid prescribing in chronic non-cancer pain: part 2–guidance. Pain Physician.

[6] Degenhardt L, Erskine H, Ferrari AJ et al (2015) Global, regional, and national incidence, prevalence, and years lived with disability for 301 acute and chronic Diseases and injuries in 188 countries, 1990–2013: a systematic analysis for the global burden of Disease Study 2013. 386(9995):743–800 The Lancet (British edition). 10.1016/S0140-6736(15)60692-4 10.1016/S0140-6736(15)60692-4 PMC4561509 26063472

[7] David D, Giannini C, Chiarelli F, Mohn A (2021). Text Neck Syndrome in Children and adolescents. Int J Environ Res Public Health.

[8] Falkenberg HK, Johansen TR, Thorud HMS (2020) Headache, eyestrain, and musculoskeletal symptoms in relation to smartphone and tablet use in healthy adolescents.

[9] Tiwari D, Goldberg A, Yorke A, Marchetti GF, Alsalaheen B (2019). Characterization of cervical spine impairments in children and adolescents post-concussion. Int J Sports Phys Therapy.

[10] Huguet A, Miró J The severity of Chronic Pediatric Pain: an epidemiological study. J pain (2008) ;9(3):226–236. 10.1016/j.jpain.2007.10.015 10.1016/j.jpain.2007.10.015 18088558

[11] Nacajauskaite O, Endziniene M, Jureniene K, Schrader H (2006). The validity of post-concussion syndrome in children: a controlled historical cohort study. Brain & Development (Tokyo 1979).

[12] Bele S, Chugh A, Mohamed B, Teela L, Haverman L, Santana MJ (2020) Patient-reported outcome measures in Routine Pediatric Clinical Care: a systematic review. Front Pead 8. 10.3389/fped.2020.00364 10.3389/fped.2020.00364 PMC7399166 32850521

[13] Lawson SL, Hogg MM, Moore CG et al Pediatric Pain Assessment in the Emergency Department: patient and caregiver agreement using the Wong-Baker FACES and the faces Pain scale–revised. Pediatric emergency care. (2019);Publish Ahead of Print. 10.1097/PEC.0000000000001837 10.1097/PEC.0000000000001837 31335787

[14] de Leeuw ED (2011) Improving Data Quality when Surveying Children and Adolescents: Cognitive and Social Development and its.

[15] Arbuckle R, Abetz-Webb L (2013). Not just little adults: qualitative methods to support the development of pediatric patient-reported outcomes. The Patient.

[16] O’Rourke D (2004). The measurement of pain in infants, children, and adolescents: from policy to practice. Phys Ther.

[17] Riley AW (2004). Evidence that School-Age Children can Self-Report on their health. Ambul Pediatr.

[18] Cleland JAPTPOCS, Childs JDPTPMBAOCS, Whitman JMPTDOCS (2008). Psychometric properties of the Neck Disability Index and Numeric Pain Rating Scale in patients with mechanical Neck Pain. Arch Phys Med Rehabil.

[19] Stupar MBDCP, Côté PDCP, Beaton DEBMP, Boyle EP, Cassidy JDDCPD (2015). Structural and construct validity of the Whiplash Disability Questionnaire in adults with acute whiplash-associated disorders. Spine J.

[20] Jacob E, Mack AK, Savedra M, Van Cleve L, Wilkie DJ (2014). Adolescent pediatric pain tool for multidimensional measurement of pain in children and adolescents. Pain Manag Nurs.

[21] Varni JW, Thompson KL, Hanson V (1987). The Varni/Thompson Pediatrie Pain Questionnaire. I. Chronic musculoskeletal pain in juvenile rheumatoid arthritis. Pain (Amsterdam).

[22] Instrument PROMIS (2022) Pediatric Pain Interference - Short Form 8a V1.0. *NIDA CTN Common Data Elements* https://cde.nida.nih.gov/instrument/0a481bfb-a67f-3c84-e050-bb89ad43314d. Accessed September 1,

[23] Shields BJ, Cohen DM, Harbeck-Weber C, Powers JD, Smith GA (2003). Pediatric Pain Measurement using a visual analogue scale: a comparison of two teaching methods. Clin Pediatr.

[24] Schifferdecker KE, Yount SE, Kaiser K (2018). A method to create a standardized generic and condition-specific patient-reported outcome measure for patient care and healthcare improvement. Qual Life Res.

[25] Organization WH (2007) International classification of functioning, disability and health: children and youth version: ICF-CY. World Health Organization. ; https://apps.who.int/iris/handle/10665/43737. Accessed September 21, 2021

[26] *International classification of functioning, disability and health. [electronic resource]: children and youth version : ICF-CY*. World Health Organization; (2007)

[27] Mokkink LB, Terwee CB, Knol DL (2010). The COSMIN checklist for evaluating the methodological quality of studies on measurement properties: a clarification of its content. BMC Med Res Methodol.

[28] Tiwari D, Clock C, Gore S, Alsalaheen B (2022). Content comparison of neck pain outcome measures using the international classification of functioning, disability and health. Int J Rehabilitation Res Int Z fur Rehabilitationsforschung Revue Int De recherches de Readaptation.

[29] Conners C. Conners CBRS™ Conners comprehensive behavior rating scales. In: Multi-Health Systems Inc. North Tonawanda, NY. Retrieved from http ?; 2008.

[30] Borgers N, de Leeuw E, Hox J (2000). Children as respondents in Survey Research: Cognitive Development and Response Quality 1. BMS.

[31] Portney LG (2020). Foundations of clinical research: applications to evidence-based practice.

[32] Okoli C, Pawlowski SD (2004). The Delphi method as a research tool: an example, design considerations and applications. Inf Manag.

[33] Zemina CL, Warren M, Yuen HK (2018). Revised Self-Report Assessment of Functional Visual performance (R-SRAFVP)-Part I: content validation. Am J Occup Therapy.

[34] Gilbert GE, Prion S (2016). Making sense of methods and measurement: Lawshe’s content Validity Index. Clin Simul Nurs.

[35] Lawshe CH (1975). A quantitative approach to content validity. Pers Psychol.

[36] Ayre C, Scally AJ (2014). Critical values for Lawshe’s content validity ratio: revisiting the original methods of calculation. Meas Evaluation Couns Dev.

[37] Ikart E (2018). Questionnaire pretesting methods: a comparison of cognitive interviewing and respondent debriefing Vis-À-Vis the study of the adoption of decision support systems by Knowledge workers. Int J Bus Inform.

[38] Scott K, Ummer O, LeFevre AE (2021). The devil is in the detail: reflections on the value and application of cognitive interviewing to strengthen quantitative surveys in global health. Health Policy Plann.

[39] Coombes L, Bristowe K, Ellis-Smith C (2021). Enhancing validity, reliability and participation in self-reported health outcome measurement for children and young people: a systematic review of recall period, response scale format, and administration modality. Qual Life Res.

[40] Vreeman RC, Nyandiko WM, Ayaya SO, Walumbe EG, Inui TS (2014). Cognitive interviewing for cross-cultural adaptation of pediatric antiretroviral therapy adherence measurement items. Int J Behav Med.

[41] Castarlenas E, Jensen MP, von Baeyer CL, Miró J (2017). Psychometric properties of the Numerical Rating Scale to Assess Self-reported Pain Intensity in Children and adolescents: a systematic review. Clin J Pain.

[42] Tsze DS, von Baeyer CL, Pahalyants V, Dayan PS (2018). Validity and reliability of the Verbal Numerical Rating Scale for Children aged 4 to 17 years with Acute Pain. Ann Emerg Med.

[43] Braun HI, Mislevy R (2005). Intuitive test theory. Phi Delta Kappan.

[44] Meadows KA (2011). Patient-reported outcome measures: an overview. Br J Community Nurs.

[45] Organization WH (2001) International Classification of Functioning, Disability and Health (ICF). ; https://www.who.int/standards/classifications/international-classification-of-functioning-disability-and-health. Accessed 3/1, 2022

[46] Tucker CA, Escorpizo R, Cieza A (2014). Mapping the content of the patient-reported outcomes Measurement Information System (PROMIS®) using the International classification of Functioning, Health and Disability. Qual Life Res.

[47] Davis LL (1992). Instrument review: getting the most from a panel of experts. Appl Nurs Res.

[48] Tilden VP, Nelson CA, May BA (1990). Use of qualitative methods to Enhance Content Validity. Nurs Res.

[49] von Baeyer CL (2006). Children’s self-reports of pain intensity: scale selection, limitations and interpretation. Pain Res Manage.

[50] Manworren RC, Stinson J (2016). Pediatric Pain Measurement, Assessment, and evaluation. Semin Pediatr Neurol.

[51] Goldstein DN, Cohn E, Coster W (2004). Enhancing participation for children with disabilities: application of the ICF enablement framework to pediatric physical therapist practice. Pediatr Phys Ther.

[52] Bell A (2007). Designing and testing questionnaires for children. J Res Nurs.

[53] Craig KD, Versloot J, Goubert L, Vervoort T, Crombez G (2010). Perceiving pain in others: automatic and controlled mechanisms. J Pain.

[54] Wiitavaara B, Björklund M, Brulin C, Djupsjöbacka M (2009). How well do questionnaires on symptoms in neck-shoulder disorders capture the experiences of those who suffer from neck-shoulder disorders? A content analysis of questionnaires and interviews. BMC Musculoskelet Disord.

[55] Chambers CT, Giesbrecht K, Craig KD, Bennett SM, Huntsman E (1999). A comparison of faces scales for the measurement of pediatric pain: children’s and parents’ ratings. Pain.

[56] Pritchard-Wiart L, Thompson-Hodgetts S, McKillop AB (2022). A multi-center, pragmatic, effectiveness-implementation (hybrid I) cluster randomized controlled trial to evaluate a child-oriented goal-setting approach in paediatric rehabilitation (the ENGAGE approach): a study protocol. BMC Pediatr.

[57] Locke EA, Latham GP (2006). New directions in goal-setting theory. Curr Dir Psychol Sci.

[58] King GA, McDougall J, Palisano RJ, Gritzan J, Tucker MA (2000). Goal attainment scaling. Phys Occup Ther Pediatr.

