# Supplementary material for: Development and validation of the activities and participation children and adolescents –neck (APCAN) measure
Source: J Patient Rep Outcomes. 2023 Oct 30;7:107. doi: 10.1186/s41687-023-00648-x (PMC10616032; doi:10.1186/s41687-023-00648-x)
Supplement: Supplementary file 1 — Supplementary Material 1 [file 41687_2023_648_MOESM1_ESM.docx]

**Plain English Summary**

1. Why is this study needed?

Given a steep rise in incidence of neck pain in children, it is crucial to systematically examine neck pain to provide best possible treatment. Currently, no age-appropriate measures exist to evaluate perceived disability due to neck pain in children and adolescents. This study provides details on development and content validity of APCAN.

1. What is the key problem/issue/question this manuscript address?

Neck pain is a rapidly growing health concern in children and can lead to significant disability if not managed in a timely manner. It is important to have valid age-appropriate measure to assess children’s perception of disability due to neck pain. This study highlights the development of a new age-appropriate patient-reported measure titled Activities and Participation Children and Adolescents-neck (APCAN) to examine perceived disability in children.

1. What is the main point of your study?

This study describes the details on how the items were selected and modified to be included in the final version of the APCAN. Additionally, by using expert consultation and using robust statistical methods it was ensured that the items are appropriate to be included in the APCAN.

1. What are your main results and what do they mean?

Overall, the APCAN demonstrated excellent content validity indicating that the items are appropriate to be used for children and are ready to be tested on a patient sample to ensure that it serves the purpose well.
