# Supplementary material for: Development and validation of the activities and participation children and adolescents –neck (APCAN) measure
Source: J Patient Rep Outcomes. 2023 Oct 30;7:107. doi: 10.1186/s41687-023-00648-x (PMC10616032; doi:10.1186/s41687-023-00648-x)
Supplement: Supplementary file 2 — Supplementary Material 2 [file 41687_2023_648_MOESM2_ESM.docx]

**Appendix 1:** Initial draft of the Activities and Participation Children and Adolescents –neck (APCAN) with ICF-CY codes

| **Item number** | **ICF CY code** | | | **Item details** | |
| --- | --- | --- | --- | --- | --- |
| 1 | s710 | | | Looking behind | |
| 2 | s710 | | | Moving neck side to side | |
| 3 | s710 | | | Moving neck up and down | |
| 4 | b140 | | | Concentrating for 30-40 minutes in classroom | |
| 5 | b134 | | | Sleeping without discomfort | |
| 6 | b780 | | | Able to move neck easily (without stiffness) | |
| 7 | b730 | | | Moving the arms | |
| 8 | d445, d520 | | | Brushing teeth, grooming | |
| 9 | d510 | | | Taking a bath/shower | |
| 10 | d210 | | | Putting on/taking off shoes | |
| 11 | d510 | | | Drying myself using a towel | |
| 12 | d540 | | | Donning/doffing clothes (shirt/t shirt/top/pants) | |
| 13 | d410 | | | Rolling over in bed | |
| 14 | d410 | | | Lying in bed | |
| 15 | d410 | | | Getting up or getting to bed | |
| 16 | d140 | | | Sitting in a chair | |
| 17 | d415 | | | Standing for a while/long time | |
| 18 | d450 | | | Walking for a while/long time | |
| 19 | d455, d410 | | | Bending down to pick up something from the floor | |
| 20 | d920 | | | Performing activities such as dancing | |
| 21 | d475 | | | Riding my bike | |
| 22 | d465 | | | Using skateboard | |
| 23 | e115, d455 | | | Climbing on a slide/playing on a play structure | |
| 24 | d920, e140 | | | Playing a musical instrument | |
| 25 | d920 | | | Playing with toys/board games/puzzles | |
| 26 | e125, d110 | | | Watching TV/movies | |
| 27 | d455 | | | Running/hopping/skipping/jumping | |
| 28 | e125, d110 | | | Playing video games | |
| 29 | d445 | | | Throwing/catching a ball | |
| 30 | d445 | | | Pulling/pushing a toy | |
| 31 | d360 | | | Talking and/or texting on the phone | |
| 32 | d166 | | | Classroom or leisure reading | |
| 33 | d410, d415 | | | Sitting in the classroom | |
| 34 | d170, d345, d155 | | | Writing/typing in class/home using books and/or tablet/laptop/desktop computer | |
| 35 | d470 | | | Riding in the school bus/car | |
| 36 | d430 | | | Carrying books/ school bag/backpacks/toys | |
| 37 | d240 | | | Taking a test/exam | |
| 38 | d155, d440 | | | Holding a pencil/crayon/fork & Knife | |
| 39 | d640 | | | Doing chores like washing dishes, using broom, vacuum | |
| 40 | d630 | | | Helping preparing meals | |
| 41 | d210, d640 | | | Making my bed/ Cleaning room | |
| 42 | d220 | | | Participate in team sports | |
| 43 | d220, d920 | | | Participating in recreational sports like swimming, gymnastics or other sporting activity of choice  **Specify:** | |
| 44 | d830, d820, d815 | | | Attending school regularly | |
| 45 | d920 | | | Keeping up with friends in the gym class | |
| 46 | d920 | | | Playing with friends on the field | |
| 47 | d810, d920 | | | Participating in arts/crafts in class | |
| 48 | d920 | | | Attending birthday parties | |
| 49 | e325, d820 | | | Maintaining relationships with friends/family/peers | |
| 50 | d760, e310 | | | Playing with siblings | |
| 51 | d650 | | | Playing with/taking care of a pet | |
| 52 | d850 | | | Doing job related duties if working in a part time/full time job | |
|  |  |  |  | |  |

Total score:
