# Supplementary material for: Development and validation of the activities and participation children and adolescents –neck (APCAN) measure
Source: J Patient Rep Outcomes. 2023 Oct 30;7:107. doi: 10.1186/s41687-023-00648-x (PMC10616032; doi:10.1186/s41687-023-00648-x)
Supplement: Supplementary file 3 — Supplementary Material 3 [file 41687_2023_648_MOESM3_ESM.docx]

**Appendix 2 – COSMIN risk of bias checklist report for face and content validity**

| **Category** | **Method utilized** |
| --- | --- |
| ***General design requirements*** | |
| Is a clear description provided of the construct to be measured? | Incidence and functional impact of neck pain in children and adolescents is described in the introduction section. |
| Is the origin of the construct clear: was a theory, conceptual framework or disease model used or clear rationale provided to define the construct to be measured? | Need to develop APCAN was identified in the introduction section. |
| Is a clear description provided of the target population for which the PROM was developed? | APCAN was targeted specifically for children and adolescents to assess activity limitation and participation restriction due to neck pain. |
| Is a clear description provided of the context of use | APCAN is focused on assessing activity limitation and participation restriction due to neck pain. |
| Was the PROM development study performed in a sample representing the target population for which the PROM was developed? | Yes. Cognitive interviews were conducted for children ranging between 8-18 years. |
| Was an appropriate qualitative data collection method used to identify relevant items for a new PROM? | Comprehensive literature search along with the review of the ICF-CY model was performed to identify relevant items for APCAN. |
| Were skilled group moderators/interviewers used? | Research team members who were experienced in conducting cognitive interviews, conducted the interviews. |
| Were the group meetings or interviews based on an appropriate topic or interview guide? | Yes. A semi-structured interview guide was used to conduct the cognitive interviews. |
| Were the group meetings or interviews recorded and transcribed verbatim? | Yes. Interviews were recorded using digital audio recorder, transcribed and deidentified. |
| Was an appropriate approach used to analyze the data? | Data from the interviews was recorded and transcribed. Standardized measurement i.e., Content validity ratio and content validity index were used to establish content validity. |
| Was at least part of the data coded independently? | Two research team members independently coded the data |
| Was data collection continued until saturation was reached? | Yes. Data saturation point was reached after two rounds of cognitive interviews. |
| ***Cognitive interview*** | |
| Was a cognitive interview study or other pilot test conducted? | Yes. Two rounds of cognitive interviews were performed with children between 8-18 years of age. |
| Was the cognitive interview study or other pilot test performed in a sample representing the target population? | Yes. Two rounds of cognitive interviews were performed with children between 8-18 years of age. |
| Were patients asked about the comprehensibility of the PROM? | Children were asked questions related to comprehensiveness of the measure. |
| Were all items tested in their final form? | A second round of cognitive interview was conducted with the final version of the measure. No areas of further modifications were identified. |
| Was an appropriate qualitative method used to assess the comprehensibility of the PROM instructions, items, response options, and recall period? | Yes. A semi-structured cognitive interview method was used to assess comprehensibility of the PROM instructions, items and response options. |
| Was each item tested in an appropriate number of patients? | Yes. Each item was tested in 6 children between the age of 8-18 years. |
| Were skilled interviewers used? | Research team members who were experienced in conducting cognitive interviews, conducted the interviews. |
| Were the interviews based on an appropriate interview guide? | Yes. A semi-structured interview guide was used to conduct the cognitive interviews. |
| Were the interviews recorded and transcribed verbatim? | Yes. Interviews were recorded using digital audio recorder, transcribed and deidentified. |
| Was an appropriate approach used to analyze the data? | Each item was scrutinized and revised based on the results from the cognitive interviews. |
| Were at least two researchers involved in the analysis? | Yes. Two researchers reviewed the data extracted from the cognitive interviews. |
| Were problems regarding the comprehensibility of the PROM instructions, items, response options, and recall period appropriately addressed by adapting the PROM? | Each item was scrutinized and revised to improve comprehensibility and response options based on the results from the cognitive interviews. |
| Were patients asked about the comprehensiveness of the PROM? | Children were asked questions related to comprehensiveness of the measure. |
| Was the final set of items tested? | A second round of cognitive interview was conducted with the final version of the measure. No areas of further modifications were identified. |
| ***Asking patients about relevance, comprehensiveness, and analyses methods*** | |
| Was an appropriate method used to ask patients whether each item is relevant for their experience with the condition? | During cognitive interviews, children were asked if they felt whether each item is important to them and is relevant. Also, children were provided an opportunity to add items which they felt were important and were not already in the measure. |
| Was each item tested in an appropriate number of patients? | Yes. Each item was tested in 6 children between the age of 8-18 years. |
| Were skilled interviewers used? | Research team members who were experienced in conducting cognitive interviews, conducted the interviews. |
| Were the group meetings or interviews based on an appropriate topic or interview guide? | Yes. A semi-structured interview guide was used to conduct the cognitive interviews. |
| Were the group meetings or interviews recorded and transcribed verbatim? | Yes. Interviews were recorded using digital audio recorder, transcribed and deidentified. |
| Was an appropriate approach used to analyze the data? | Each item was scrutinized and revised based on the results from the cognitive interviews. |
| Were at least two researchers involved in the analysis? | Yes. Two researchers reviewed the data extracted from the cognitive interviews. |
| *Asking professionals about relevance, comprehensiveness and analysis* | |
| Was an appropriate method used to ask professionals whether each item is relevant for the construct of interest? | Yes. A modified-Delphi process was utilized to ask professionals about the relevance of items. |
| Were professionals from all relevant disciplines included? | Physical therapists, occupational therapists and nurses were included as part of the expert panel. |
| Was each item tested in an appropriate number of professionals? | Ten professionals were included in the modified Delphi process. |
| Was an appropriate approach used to analyse the data? | Cutoff scores based on Lawshe’s Content Validity Ratio was used to retain items and Content validity index were used to examine overall content validity. |
| Were at least two researchers involved in the analysis? | Two research team members were involved in the analysis. |
