# Supplementary material for: Development and validation of the activities and participation children and adolescents –neck (APCAN) measure
Source: J Patient Rep Outcomes. 2023 Oct 30;7:107. doi: 10.1186/s41687-023-00648-x (PMC10616032; doi:10.1186/s41687-023-00648-x)
Supplement: Supplementary file 4 — Supplementary Material 4 [file 41687_2023_648_MOESM4_ESM.docx]

Authors Response to Reviewers

Response to Reviewer #1

| Comments | Authors’ response |
| --- | --- |
| Abstract  - "The purpose of this study was to develop and establish the content validity of a new measure i.e., Activities and Participation Children and Adolescents -neck (APCAN), measure specific to evaluating activity limitation and participation restrictions in children." Says measure twice | Thank you for the note. As suggested, we have revised the sentence. The section now reads as:  Hence, the purpose of this study was to develop a new measure (i.e., Activities and Participation Children and Adolescents -neck [APCAN]) specific to evaluating activity limitation and participation restrictions in children and adolescents and to establish its content validity. |
| - Methods: specify who the interviews were with, also do you mean "cognitive debriefing"? | Thank you for this comment. We have changed “cognitive testing” to “cognitive interviewing” throughout the manuscript.  We are using the term “cognitive interviewing” defined in the literature as a qualitative research method to improve the validity of quantitative surveys. During this process the participants reported on their internal cognitive process used to answer the survey questions. We asked them to think out loud and describe how they interpreted the survey question and formulated a response. There were some probing questions about the meaning of specific terms. We believe that cognitive interviewing differs from respondent debriefing which focuses on post-survey reflections and often used later in the survey development process at times to gather information before the next administration of a continuing survey.  We have added the following references to clarify our process:  Ikart EM. Questionnaire Pretesting Methods: A Comparison of Cognitive Interviewing and Respondent Debriefing Vis-à-vis the Study of the Adoption of Decision Support Systems by Knowledge Workers. *International Journal of Business and Information.* 2018;13(2):119-154. doi:10.6702/ijbi.201806_13(2).0001  Scott K, Ummer O, LeFevre AE. The devil is in the detail: reflections on the value and application of cognitive interviewing to strengthen quantitative surveys in global health. Health Policy Plan. 2021;36(6):982-995. doi:10.1093/heapol/czab048 |
| Background  - "Additionally, neck pain results in increased missed school days, poor academic performance, and poor sleep, resulting in negative health behaviors."—what negative health behaviors in particular? | Thank you for this comment.  We have elaborated on this statement:  “Additionally, neck pain results in increased missed school days, poor academic performance, and poor sleep, resulting in negative health behaviors,^10^ such as being more irritable and feeling alienated.”  Reference:  Fares J, Fares M, Fares Y. Musculoskeletal neck pain in children and adolescents: Risk factors and complications. Surgical neurology international. 2017;8(1):72. doi:10.4103/sni.sni_445_16 |
| - "Although there are some pediatric measures such as the Adolescent Pediatric Pain Tool,21 Pediatric Pain Questionnaire,22 and The National Institutes of Health (NIH) Patient Reported Outcomes Measurement Information System (PROMIS) pediatric pain interference scale23 that focus on pain assessment in children and youth."—remove the "Although" that begins the sentence | Thank you for highlighting this. We have removed the word “although”. The sentence now reads as;  There are some pediatric measures such as the Adolescent Pediatric Pain Tool,^21^ Pediatric Pain Questionnaire,^22^ and The National Institutes of Health (NIH) Patient Reported Outcomes Measurement Information System (PROMIS) pediatric pain interference scale^23^ |
| - Have any of these other PROMs been used to assess neck pain in other studies? Either in adults or children. Add this info. | Thank you for the note. While these are generic measures, none of these measures have been used to assess neck pain in children specifically. We have addressed this in the introduction section:  There are some pediatric measures such as the Adolescent Pediatric Pain Tool, Pediatric Pain Questionnaire, and The National Institutes of Health (NIH) Patient Reported Outcomes Measurement Information System (PROMIS) pediatric pain interference scale that focus on pain assessment in children and youth. However, these measures are generic, not specific to neck pain, do not cover the interaction of how the pain restricts participation and limits activities across multiple environments, and have not been used to evaluate neck pain in this population. |
| - It's not clear why existing PROMs weren't modified to address neck pain, rather than creating an entirely new PROM. Please address either here or in the methods | Thank you for this note. While we agree that modifying existing measures would have been a good solution, the existing measures for children were designed as generic measures and were meant to be nonspecific to a particular region. Secondly, existing measures for the adult population could not be adapted to a modified version as these measures were not built using the ICF- CY framework and were designed for the adult population. The ICF-CY model recommends that “The manifestations of disability and health conditions in children and adolescents are different in nature, intensity, and impact from those of adults. These differences need to be considered so that classification of content is sensitive to the changes associated with development and encompasses the characteristics of different age groups and environments”. Given the extensive nature of changes needed in existing measures, modification/adaptation was not relevant. |
| - "Hence, the purpose of this study was to develop a new measure i.e., Activities and Participation Children and Adolescents -neck (APCAN), measure specific to evaluating activity limitation and participation restrictions in children and adolescents and to establish its content validity."—change to "Hence, the purpose of this study was to develop a new measure (i.e., Activities and Participation Children and Adolescents -neck [APCAN]) specific to evaluating activity limitation and participation restrictions in children and adolescents and to establish its content validity." | Thank you for the note. We have changed the sentence. The section now reads as:  Hence, the purpose of this study was to develop a new measure (i.e., Activities and Participation Children and Adolescents -neck [APCAN]) specific to evaluating activity limitation and participation restrictions in children and adolescents and to establish its content validity. |
| Methods  - Same comment as abstract—do you mean cognitive debriefing vs. testing? | Thank you for this question.  We have changed “cognitive testing” to “cognitive interviewing” to help distinguish between cognitive interviewing and respondent debriefing. |
| - As you note in the methods there are other neck pain measures, so the rationale for creating a new one from scratch is not clear. Why could an existing measure not be modified to be applicable to children and adolescents? | Thank you for this note. While we agree that modifying existing measures would have been a good solution, the existing measures for children were designed as generic measures and were meant to be nonspecific to a particular region. Secondly, existing measures for the adult population could not be adapted to a modified version as these measures were not built using the ICF- CY framework and were designed for the adult population. The ICF-CY model recommends that “The manifestations of disability and health conditions in children and adolescents are different in nature, intensity, and impact from those of adults. These differences need to be considered so that classification of content is sensitive to the changes associated with development and encompasses the characteristics of different age groups and environments”. Given the extensive nature of changes needed in existing measures, modification/adaptation was not relevant. |
| - Not sure all the details of the linkage to ICF-CY are necessary | Thank you for highlighting this. As suggested, we have simplified this section and have made it concise. The section now reads as: Two experts from the study team linked each item from the APCAN to the ICF-CY categories. ICF-CY follows a classification system that is organized based on hierarchy and inter-relatedness of levels. The ICF-CY consists of a section on functioning and disability that includes body functions and structures and activities and participation, and a section on contextual factors that includes environmental and personal factors. |
| - I think you mean cognitive debriefing vs. cognitive testing—please clarify | Thank you for this question. Please see response above. We have changed “cognitive testing” to “cognitive interviewing” to help distinguish between cognitive interviewing and respondent debriefing. |
| - How long ago did the children have neck pain who participated in the cognitive debriefing? There may be differences in those currently experiencing it vs. those who had it in the past—were any differences noted? | Thank you for this question. We have added a table which includes participant characteristics. We did not note any differences between the participant experiencing neck pain at the time of the study and those with a remote history of neck pain. Please see table 2 |
| - Where were these children recruited from? Was any compensation given for their time? | Participants were recruited via social media and from parent groups on the researchers’ social media accounts. Participants received a $15 gift card for their participation.  We have added the following text to the manuscript to detail this process.  “Children and adolescents aged 8-18 years old who speak English as a primary language and currently experiencing neck pain or with a history of neck pain were recruited via a flyer posted to the researchers’ social media accounts.”  “Participants were provided with a $15 gift card for participating.” |
| - Only two rounds of cognitive debriefing interviews with 6 children total is quite small—how can you be sure there is face validity with this sample size? | Thank you for this comment. Here is the rationale for concluding cognitive interviews after two rounds.  While a wide range of participant numbers can be potentially recruited for cognitive interviewing, it has been recommended in literature that given the iterative nature of this methodology, the number of respondents should be decreased/ stopped as the survey questions become more appropriate to the context and population under study. (Scott et al.)  As the reviewer notes, all items were revised after the first round of cognitive interviews. However, after interviews # 4 and 5, only three items required revision and clarification (items 5, 6, and 11). For example, for item 6, only the word “top” was deleted from the question. For item 10 only one word was added to the following survey item: “I can play with toys/board  games/puzzles/do arts, crafts while  sitting on a chair/floor like I usually  do.” The only change that was made was that “arts, crafts” was changed to “arts and crafts.”  Due to these small changes to survey items after interview with participant number 6, no further changes were warranted, the researchers made the decision to stop cognitive interviews. We believe that the small changes to only 3 items after round two of cognitive testing provides face validity.  Reference  Scott K, Ummer O, LeFevre AE. The devil is in the detail: reflections on the value and application of cognitive interviewing to strengthen quantitative surveys in global health. *Health Policy Plan.* 2021;36(6):982-995. doi:10.1093/heapol/czab048 |
| Results  - Content mapping was done but why was an exploratory factor analysis not done? This would strengthen the validity | Thank you for highlighting this. We agree that it is important to perform factor analysis. We are currently in the process of data collection for establishing the factor structure, construct validity, internal consistency, and reliability of APCAN. We have added this as a suggestion for further research in the discussion section. The segment now reads as: Finally, further research on examining factor structure using exploratory factor analysis, establishing internal consistency and construct validity using Rasch analysis are needed to strengthen the APCAN. |
| - During the first round of cognitive debriefing all 20 items were revised for clarity—this makes me further question whether 6 interviews is sufficient. Though you say item revision was no longer required how can you be certain if a third round was not done on the revised items? If only the last participant demonstrated understanding of all items, I do not think you can claim face validity | Thank you for this comment. Here is the rationale for concluding cognitive interviews after two rounds.  While a wide range of participant numbers can be potentially recruited for cognitive interviewing, it has been recommended in literature that given the iterative nature of this methodology, the number of respondents should be decreased/ stopped as the survey questions become more appropriate to the context and population under study. (Scott et al.)  As the reviewer notes, all items were revised after the first round of cognitive interviews. However, after interviews # 4 and 5, only three items required revision and clarification (items 5, 6, and 11). For example, for item 6, only the word “top” was deleted from the question. For item 10 only one word was added to the following survey item: “I can play with toys/board  games/puzzles/do arts, crafts while  sitting on a chair/floor like I usually  do.” The only change that was made was that “arts, crafts” was changed to “arts and crafts.”  Due to these small changes to survey items after interview with participant number 6, no further changes were warranted, the researchers made the decision to stop cognitive interviews. We believe that the small changes to only 3 items after round two of cognitive testing provides face validity.  Reference  Scott K, Ummer O, LeFevre AE. The devil is in the detail: reflections on the value and application of cognitive interviewing to strengthen quantitative surveys in global health. *Health Policy Plan.* 2021;36(6):982-995. doi:10.1093/heapol/czab048 |
| - Where are tables describing the characteristics of the experts and the children? This is key to interpreting the results and is not mentioned at all besides the age ranges of the children | Thank you for this comment. A table including characteristics of the children who participated in cognitive interviewing has been added. Please see Table 2. |
| Discussion  - Why were Likert scale responses not chosen? | Thank you for the note. We agree that although Likert scales are frequently used in patient reported outcome measures, there are some limitations when it comes to children. We have added some details about this in the discussion section. The segment now reads as:  Previous studies have highlighted that children often experience difficulty in interpreting the middle point (e.g. moderate) on Likert ratings. ^38^Additionally, children have shown to demonstrate difficulty in quantifying the differences between response options (e.g. occasionally, almost never). On the other hand, Numeric rating scale (ranging from 0-10) has been well-established as a reliable and valid measure of pain intensity in children and adolescents.    References:  Coombes L, Bristowe K, Ellis-Smith C, et al. Enhancing validity, reliability and participation in self-reported health outcome measurement for children and young people: a systematic review of recall period, response scale format, and administration modality. Qual Life Res*.* (2021);30(7):1803-1832. <https://doi.org/10.1007/s11136-021-02814-4>  Vreeman RC, Nyandiko WM, Ayaya SO, Walumbe EG, Inui TS. Cognitive interviewing for cross-cultural adaptation of pediatric antiretroviral therapy adherence measurement items. International journal of behavioral medicine*.* (2014);21(1):186-196. <https://doi.org/10.1007/s12529-012-9283-9>  Castarlenas E, Jensen MP, von Baeyer CL, Miró J. Psychometric Properties of the Numerical Rating Scale to Assess Self-Reported Pain Intensity in Children and Adolescents: A Systematic Review. Clin J Pain*.* (2017);33(4):376-383. <https://doi.org/10.1097/ajp.0000000000000406> |
| - "For e.g., a child's overall score on the APCAN of 10 out of 210"—I thought the maximum score was 200?  - What are the different domains? List in the methods? | Thank you for highlighting this typo. The total score is out of 200. We have corrected this error. The sentence now reads as:  For e.g., a child’s overall score on the APCAN of 10 out of 200, provides an initial indication that the overall severity of the impact of neck pain on activity and participation is potentially low |
| - The methods were good, however the small sample size for the interviews is a key point that isn't appropriately addressed and calls into question the content validity | Thank you for this comment. Here is the rationale for concluding cognitive interviews after two rounds.  While a wide range of participant numbers can be potentially recruited for cognitive interviewing, it has been recommended in literature that given the iterative nature of this methodology, the number of respondents should be decreased/ stopped as the survey questions become more appropriate to the context and population under study. (Scott et al.)  As the reviewer notes, all items were revised after the first round of cognitive interviews. However, after interviews # 4 and 5, only three items required revision and clarification (items 5, 6, and 11). For example, for item 6, only the word “top” was deleted from the question. For item 10 only one word was added to the following survey item: “I can play with toys/board  games/puzzles/do arts, crafts while  sitting on a chair/floor like I usually  do.” The only change that was made was that “arts, crafts” was changed to “arts and crafts.”  Due to these small changes to survey items after interview with participant number 6, no further changes were warranted, the researchers made the decision to stop cognitive interviews. We believe that the small changes to only 3 items after round two of cognitive testing provides face validity.  Reference  Scott K, Ummer O, LeFevre AE. The devil is in the detail: reflections on the value and application of cognitive interviewing to strengthen quantitative surveys in global health. *Health Policy Plan.* 2021;36(6):982-995. doi:10.1093/heapol/czab048 |
| - Since all the items were linked to the ICF-CY this makes an EFA of more significance to see what items group together and whether they follow the mapping to the ICF-CY | Thank you for this comment. We agree that it is important to examine the factor structure of APCAN to ensure that the items group together and follow ICF-CY mapping. We are working towards this and will be disseminating the findings in another manuscript. We are currently in the process of data collection for establishing the factor structure, construct validity, internal consistency, and reliability of APCAN. We have added this point as a suggestion for further research in the discussion section. The segment now reads as:  Finally, further research on examining factor structure using exploratory factor analysis, establishing internal consistency and construct validity using Rasch analysis are needed to strengthen the APCAN. |
| - "Existing outcomes measures that do assess neck pain were not designed specifically for the child and adolescent population and do not use the ICF-CY model.49 Additionally, these measures show a misalignment between the symptoms expressed by the patients with neck pain and the content of the questionnaires."—this should be in the intro, it answers my previous comments on the rationale for creating a new PROM, though some additional comments on why existing items could not be modified should still be added | Thank you for this comment. The following change has been made:  Future studies to develop a computerized adaptive version that self-selects items based on responses could be helpful in reducing the administration of the measure. |
| - Under limitations, typo: "Future studies reviewto develop…" | Thank you for the note. We have corrected the typo. The sentence now reads as:  Future studies to develop a computerized adaptive version that self-selects items based on responses could be helpful in reducing the administration of the measure |
| - Under future directions, why Rasch and not factor analysis? | Thank you for this note. We are currently in the process of establishing the factor structure (exploratory and confirmatory factor analysis – EFA and CFA) which is the first step in Rasch analysis. As part of Rasch analytic methods, we will also extend CFA finds to then determine item difficulty levels using Wright maps. We have added this as a suggestion for further research in the discussion section. The segment now reads as: Finally, further research on examining factor structure using exploratory factor analysis, establishing internal consistency and construct validity using Rasch analysis are needed to strengthen the APCAN. |
| - Limitations does not mention the small sample size for the cognitive debriefing interviews with children | While the sample size was adequate and reached saturation to attain validity in the current sample of children who speak English as a primary language, we agree that we could have utilized a diverse sample with children who do not have English as their primary language The following was added to the limitations section:  Cognitive interviewing was limited to children who speak English as a primary language. Future research should include translating and cross-culturally adapting the neck pain measure to other languages. |
| Table 1  - Rather than noting the ICF-CY code it would be more useful to write out the domain, etc. | Thank you for the suggestion. We have revised the tables and included ICF domain instead of categories. Please see tables. |
| - Table 1 could be moved to supplementary—readers will be more interested in the final items | Thank you for the suggestion. We agree that the readers would be more interested in the final items with their respective ICF domains. We have revised tables and have included only the final items. Initial list of items is now labelled as Appendix 1. |

Reviewer 2

| Comments | Authors’ response |
| --- | --- |
| Background, page 2, line 47 - The sentence ending with reference #25. Recommend addition of the word "may" to read as Compared to generic measures, condition-specific measures "may" provide better. As currently written, it sounds like an absolute. | Thank you for highlighting this. We agree that addition of the word “may” will make the sentence better. We have made this addition. The sentence now reads as:  Compared to generic measures, condition-specific measures may provide better relevance and responsiveness and demonstrate the potential to differentiate patient groups by clinically predominant symptoms or health concerns. |
| Reference 25 is a study that takes well-established measures from PROMIS and organizes them to condition. I know what the authors want to say, yet I caution against such a statement of certainty. | Thank you for the note. We agree that this fact can not be stated with certainty. Hence, as suggested in a previous comment, we have added the word “may” to remove certainty. The sentence now reads as:  Compared to generic measures, condition-specific measures may provide better relevance and responsiveness and demonstrate the potential to differentiate patient groups by clinically predominant symptoms or health concerns. |
| Page 9, "Face and Content Validity of APCAN" - I recommend the authors frame their discussion around the items in box D of the COSMIN checklist (see reference 28)measure. I believe they can answer each of the five questions in box D. This is Box 2 at www.cosmin.nl - see risk of bias checklist | Thank you for this note. As recommended, we have created a table that describes the procedures utilized based on COSMIN checklist for risk of bias. Please see attached appendix #2  We also added reference to COSMIN in the discussion which reads as:  Additionally, COSMIN guidelines were followed while developing and establishing the content validity (Appendix 2).^27^ Based on COSMIN recommendations, good face and content validity were attained through a thorough review of the ICF-CY model, a review of existing measures specific to neck pain, inclusion of experts from multiple disciplines that closely work with patients with pediatric neck pain, and the use of a systematic process of obtaining stakeholder input to revise and refine the measure (Appendix 2). |
| Page 10, "Comprehensiveness" - I recommend the authors also frame the discussion around the COSMIN risk of bias instrument | Thank you for this note. We added this to the discussion section which now reads as:  The APCAN was formed to address all components of COSMIN recommendations to ensure comprehensiveness, including use of appropriate qualitative data, skilled interviewers, use of interview guides, and interviewing till attainment of data saturation. Appendix 2 highlights specific details on the use of COSMIN to ensure comprehensiveness. |
| In summary - Recommend a table illustrating how the authors' PROM Development, Content, and Structural Validity followed (or didn't) follow the COSMIN recommendations. | Thank you. As recommended, we created a table for COSMIN. Please see Appendix 2 |
| In the section - "Limitations and future directions," - Wording of the second sentence looks like a typo. Please replace "reviewto" with the correct wording. | Thank you for this comment. The following change has been made:  Future studies to develop a computerized adaptive version that self-selects items based on responses could be helpful in reducing the administration of the measure. |
